# Repeated mild traumatic brain injury impairs fracture healing in male mice

**DOI:** 10.1186/s13104-022-05906-7

**Published:** 2022-01-29

**Authors:** Chandrasekhar Kesavan, Charles Rundle, Subburaman Mohan

**Affiliations:** 1 Musculoskeletal Disease Center, VA Loma Linda Healthcare System, 11201 Benton Street, Loma Linda, CA 92357 USA; 2 Departments of Medicine, Loma Linda University, 11234 Anderson St, Loma Linda, CA 92354 USA; 3 Orthopedic Surgery, Loma Linda University, 11234 Anderson St, Loma Linda, CA 92354 USA

**Keywords:** r**-**mTBI, Bone fracture, Mice, Three-point bending, Micro-CT

## Abstract

**Objectives:**

The goal of this study was to evaluate the long-term impact of repeated (r) mild traumatic brain injury (mTBI) on the healing of fractures in a mouse model. Ten week-old male mice were subjected to r-mTBI once per day for 4 days followed by closed femoral fracture using a three-point bending technique, 1 week post impact and fracture healing phenotype evaluated at 20 weeks of age.

**Results:**

Micro-CT analysis of the fracture callus region at nine weeks post fracture revealed reduced bone volume (30%, *p* < *0.05*) in the r-mTBI fracture group compared to the control-fracture group. The connectivity density of the fracture callus bone was reduced by 40% (*p* < *0.01)* in the r-mTBI fracture group. Finite element analysis of the fracture callus region showed reduced failure load (*p* = *0.08*) in the r-mTBI group compared to control group. There was no residual cartilage in the fracture callus region of either the r-mTBI or control fracture group. The reduced fracture callus bone volume and mechanical strength of fracture callus in r-mTBI mice 9 weeks post fracture are consistent with negative effects of r-mTBI on fracture healing over a long-term resulting in decreased mechanical strength of the fracture callus.

## Introduction

Traumatic brain injury (TBI) is an acute injury that occurs as a result of an external force caused by falls, accidents, sports injuries and military conflicts [1, 2] and can be classified into three forms: mild, moderate, and severe. A Glasgow Coma Scale score of 13 to 15 is considered a mild injury, 9 to 12 is considered a moderate injury, and 8 or less is considered a severe injury [3]. Epidemiological studies have shown that typically 10% of TBI injuries are severe and patients are hospitalized, 10% are moderate TBI and approximately 80% of injuries are mTBI [4] without hospitalization.

The mTBI is common among professionals in collision sports and in military personnel. While a single impact may not always result in clinical manifestations, repeated injuries can promote progressive damage to the brain and lead to functional impairments to other parts of the body over time [5–7]. It is also reported that a history of r-mTBI might place people at increased risk for developing PTSD (https://www.brainline.org/article/tbi-and-ptsd-navigating-perfect-storm), thus suggesting r-mTBI is as important as severe TBI and therefore understanding how r-mTBI affects different tissues in the body could lead to identification of therapeutic strategies to reduce the pathophysiological conditions caused by r-mTBI in humans.

Reports have shown that patients with any type of TBI have hypothalamus-pituitary dysfunction [8, 9]. In response to trauma, the function of the hypothalamus-pituitary axis (HPA) is disrupted, causing an alteration in the secretion of hormones by the pituitary gland. The most common manifestation of HPA dysfunction due to TBI is reduced growth hormone (GH) production, a key regulator of bone metabolism that mediates its anabolic effects directly or indirectly through insulin-like growth factor (IGF)-I [10–12]. By using a mouse model of mTBI, we have demonstrated that r-mTBI decreased trabecular bone formation and that this reduction was in part due to a deficiency in the GH-IGF-I axis [13]. However, at cortical bone sites, the bone mass reduction due to r-mTBI was minimal, possibly due to reduced bone turnover at this site compared to trabecular bone sites during normal physiology.

A number of clinical and preclinical studies have been published assessing the impact of brain injury caused by motor-vehicle accidents, combat injuries, on fracture healing. However, the outcomes of these studies have been mixed as some have shown accelerated fracture healing while others have failed to establish a positive correlation between TBI and fracture healing [14–18]. Past studies in mice have utilized controlled cortical impact or closed femur head injury models to evaluate the impact of single brain injury on fracture healing at 3–4 weeks post-impact [17, 19]. Although r-mTBI is more frequent with long-term functional deficits [20], the effect of r-mTBI on fracture healing has not been established. Based on our findings that r-mTBI mice exhibited GH deficiency over a long term and that the GH/ IGF-I axis plays a critical role in regulating skeletal development, we tested the prediction that r-mTBI is associated with impaired healing of fractured bone over a long-term.

## Main text

### Methods

#### Animals

Nine-week old male C57BL/6 J mice were purchased from the Jackson Laboratory (Bar Harbor Maine) and were housed for 1 week to acclimate under standard conditions prior to experiments Ten week old mice were randomly divided into two groups (impacted and non-impacted control) based on their body weight (n = 10 mice per group).

#### r-mTBI in mice

**Fig. 1:**
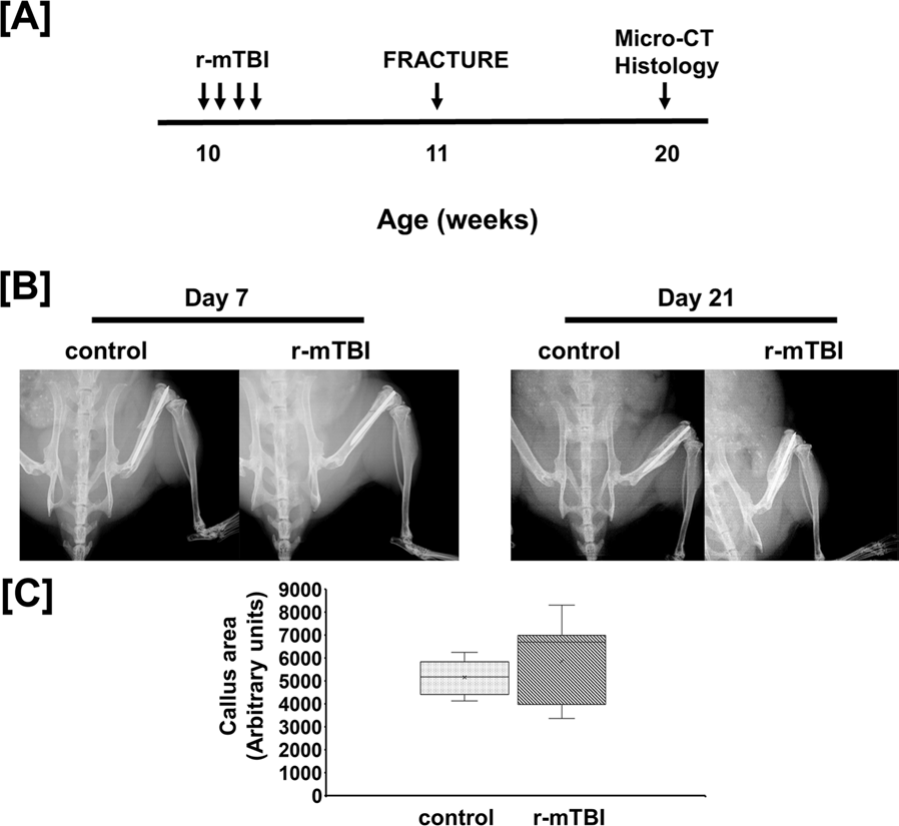
**A** The study design and **B** Day 7 and 21 post fracture femur X-ray images and **C** semi-quantitative analysis of the fractured callus from day 21 of control and r-mTBI mice. The red arrow shows callus area

Mice were subjected to r-mTBI by using a 75 g directed weight dropped on the skull from a height of 1.5 m once per day for four consecutive days, under isoflurane anesthesia (5% Isoflurane and 95% oxygen) once per day for 4 consecutive days as described previously [13, 21]. The non-impacted anesthetized mice were used as control (Fig. 1A).

#### Closed femoral fracture

At 11 weeks of age, a closed femur fracture was performed using the Instron servo hydraulic tester. Analgesia (buprenorphine, 50 µg/kg in 0.05 ml saline) was given intraperitoneally approximately every 12 h during the day after surgery for 2 days or until there was no observed pain or discomfort [22, 23].

#### Faxitron

Femur X-rays of anesthetized mice were obtained from Faxitron Radiography system (Hologic, USA) at day 7 and 21 post fracture using 20 kV X-ray energy for 10 s. Fiji an open-source software was used to analyze the fracture callus semi-quantitatively. Briefly, the JEPG or TIFF images were converted to 8 bits with gaussian blur at 1.0. A rectangular box was placed at the callus site and scaled. Using the freeform tool in the software, the areas of the callus on either side of the pin were outlined and all data were analyzed.

#### Micro-CT analysis

At 20 weeks of age, mouse was euthanized using carbon dioxide method for 3–5 min. Femurs were collected from the euthanized mice and fixed in 10% formalin for 72 h. The intramedullary pin was carefully removed from the fractured femurs prior to micro-CT. Both fractured and non-fractured femurs were scanned at a resolution of 10.4 microns at 55 kilovoltage peak (kVp) X-ray energy [13] and images were reconstructed using the 2-D image software provided by Scanco Viva-CT 40 instrument (Scanco, USA, Wayne, PA). The entire fracture callus of approximately 500 slices was contoured for analysis using 220–570 mg/cm^3^ and 570–1000 mg/cm^3^ density threshold to capture the low-density immature and fully mineralized remodeled bone, respectively. Bone parameters, such as tissue volume (TV, mm^3^), bone volume (BV, mm^3^), BV/TV (%) and connective density were measured in the fracture callus area using the Scanco analysis software.

#### Finite element (FE) analysis

The FE models of the fracture callus were created directly from the segmented micro-CT images by converting bone tissue voxels in the scanned volume of interest (VOI) to 8 node brick elements. Boundary conditions were set to simulate a high-friction compression test in the bone axial direction. Based on the results of the simulation, the stiffness S (Newton (N)/milli meter (mm)) and estimated failure load (N) were calculated. The failure load estimation was based on the 'Pistoia criterion' but modified by taking the von Mises stress as the parameter and setting its critical value to 70 MPa. For the Young's modulus chosen here, the latter stress value corresponds to the 0.7% strain used in the original criterion such that results are comparable [24, 25]. The stiffness, failure data and connectivity density are presented as ratio which is obtained by dividing each right femur fracture callus data with the mean of the left femur data.

#### Histology

The formalin fixed bones were washed with 1X phosphate buffered saline (PBS) and subsequently demineralized in ethylenediaminetetraacetic acid (EDTA) (14%), embedded in paraffin, and sectioned longitudinally at 5 micron per section. The bone sections were stained with Safranin-Orange to visualize cartilage [26].

#### Serum alkaline phosphatase (ALP) activity measurements

Blood was collected immediately after euthanasia and serum used for measurements of ALP activity as described [26]. The values are expressed as milliunit/milliliter (mU)/ml.

#### Statistical analysis

The Student t-test was used to compare the differences in bone parameters and serum markers between the groups. A p-value of < 0.05 was considered statistically significant. Values are presented as the mean ± standard error of mean (SEM).

## Results and discussion

**Fig.2:**
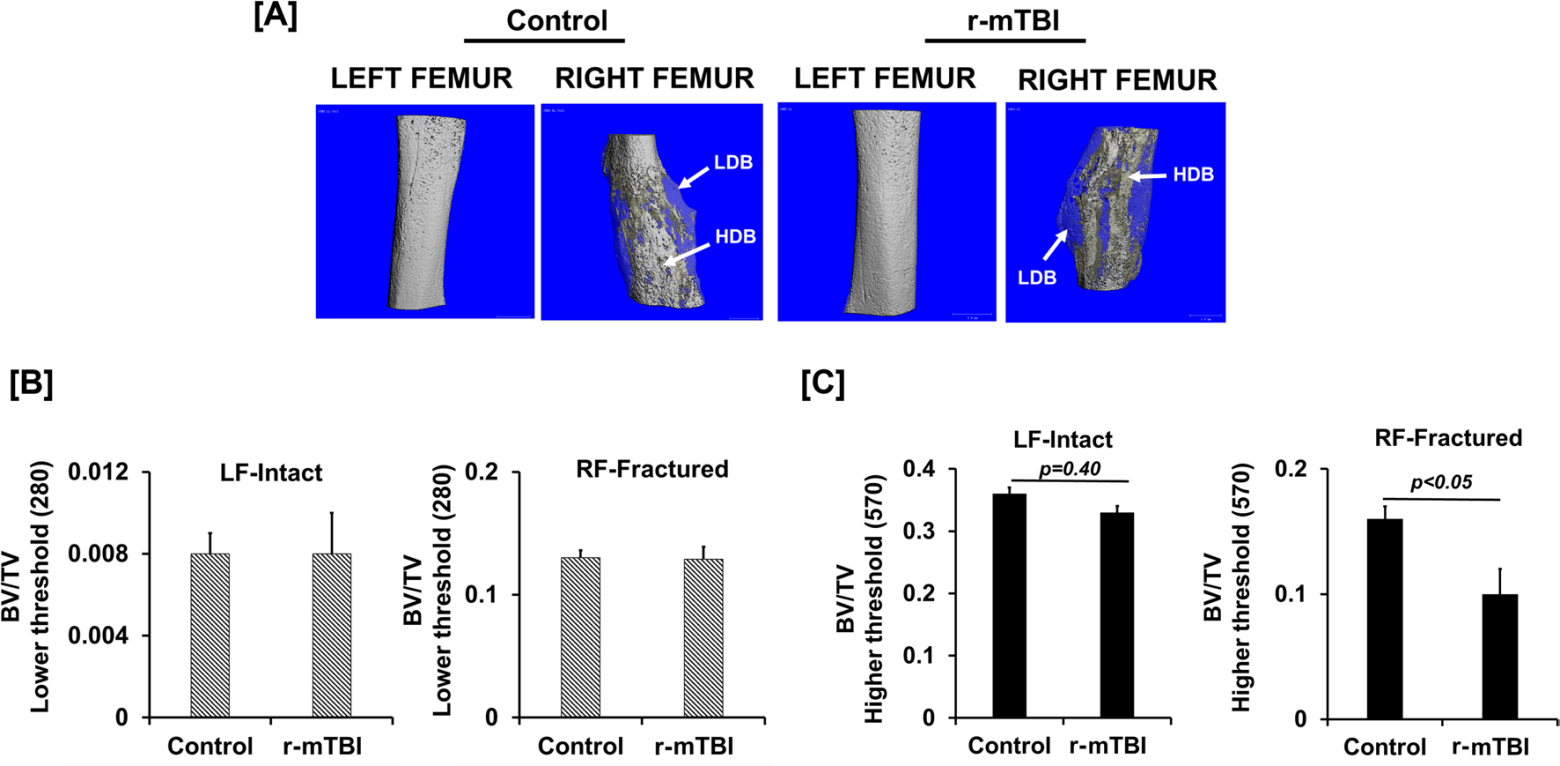
Micro-CT evaluation of fractured callus in the control and r-mTBI mice. **A** Micro-CT image showing fracture callus 9 weeks post injury in the control and r-mTBI mice. **B** Micro-CT analysis BV/TV measured at a lower threshold and **C** at a higher threshold in the non-fractured left femur (LF) and in the right femoral (RF) fracture callus of control and r-mTBI mice. Values are mean ± SEM, N = 8–10 animals/group. The arrow corresponds to lower density bone (LDB) and higher density bone (HDB)

**Fig.3:**
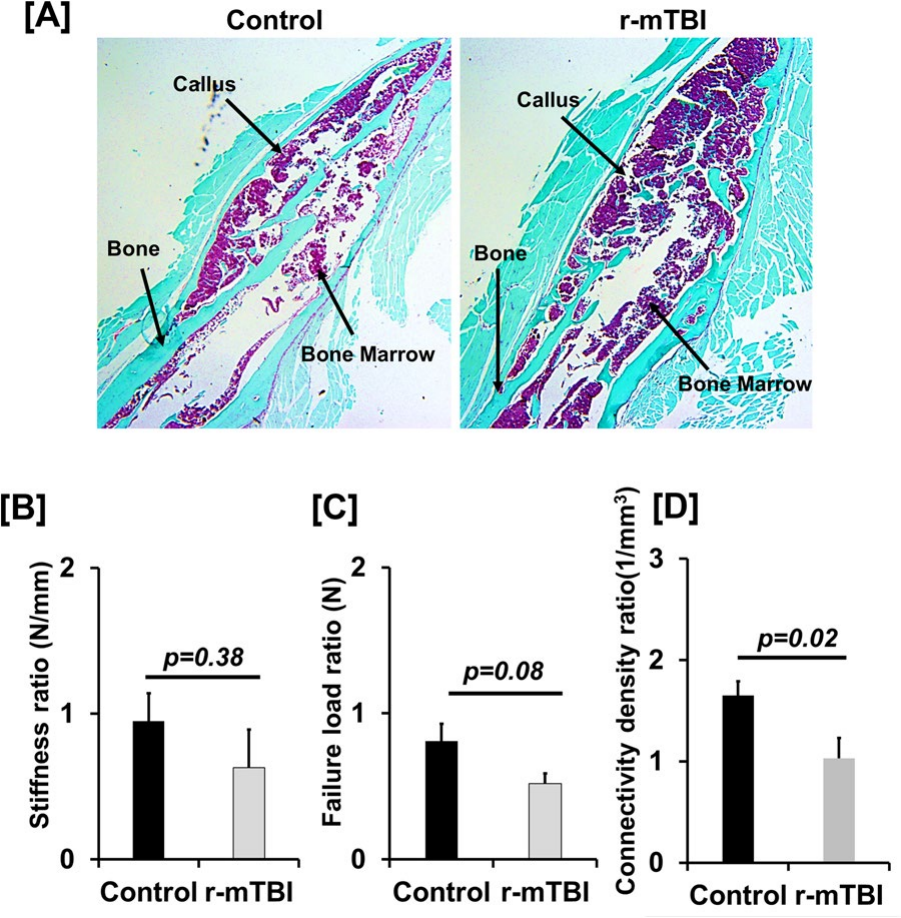
**A** Longitudinal section of fractured femur by histology and **B**–**D** fractured callus strength parameters. Sections in Fig. 3A were stained for cartilage with Safranin-O. Quantitation of **B** stiffness **C** failure load by finite element analysis and **D** connectivity density measured by micro-CT in the fracture callus of control and r-mTBI mice. Values are mean ± SEM, N = 5 animals/group for finite element analysis and N = 8/animals/group for connectivity density

TBI and skeletal fractures are frequently reported in the same patients [27, 28]. Therefore, understanding how TBI impacts bone healing is essential in promoting healing and skeletal function to improve the quality of life. Because r-mTBI has a negative effect on bone formation via a mechanism that involved the GH/IGF-I axis [13], we determined the impact of r-mTBI on healing of fractured bone in a mouse model. We used a weight drop model and a free fall mechanism to generate the brain impact without any skull fracture for inducing mTBI in mice [26]. Figure 1B shows representative X-ray image of a fractured femur at day 7 and 21 induced by a three-point bending method in the control and TBI mice. We found no significant difference in the fracture callus area between r-mTBI and control mice at day 21 post-fracturing (Fig. 1C). Figure 2A shows representative micro-CT images of fracture callus area at the femoral diaphysis of r-mTBI and control mice. Quantitative analysis revealed that there was no difference in the amount of lower density bone between control and r-mTBI mice as determined at the lower threshold setting (Fig. 2B). However, at the higher threshold setting, the BV/TV phenotype was significantly reduced in the fracture callus of r-mTBI mice (Fig. 2C) thus raising the possibility that the remodeling of immature bone into fully mineralized bone is impaired in the fracture callus of r-mTBI mice compared to control mice. To determine if cartilage to bone conversion is affected by r-mTBI, we performed safranin O staining on longitudinal sections of the right femur (Fig. 3A). There was no measurable residual cartilage present in either r-mTBI or control mice, thus suggesting that the normal cartilage to bone conversion process was complete at this time point in both groups. To determine if the decreased mineralization in the fracture callus of r-mTBI mice could result from reduced osteoblast functions, we measured serum levels of ALP activity, a measure of osteoblast function, and found a 18% reduction in activity in r-mTBI versus control mice (21.83 ± 1.84 vs. 26.04 ± 1.17 mU/ml, *p* = *0.06*)). Serum levels of ALP activity showed a positive correlation (r = 0.33, *p* = *0.16*) with fracture callus of higher threshold BV/TV and inverse correlation (r = − 0.39, *p* = *0.09*) with lower threshold of BV/TV in the r-mTBI and control mice. Taken together, these data suggest that r-mTBI has a negative effect on fracture healing.

Since the fracture callus of r-mTBI mice show a reduced amount of fully mineralized bone compared to control mice and since mineralization is an important predictor of bone strength, we predicted that r-mTBI has a negative impact on the mechanical properties of newly formed bone in the fracture callus. To test this, we estimated the mechanical properties of the fracture callus of r-mTBI and control mice using the FE analysis program from Scanco. Although the stiffness (*p* = *0.38*) was not significantly different, failure load, a measure of bone breaking strength, were reduced by 30–40% (*p* = 0.08) in the fracture callus of r-mTBI mice (Fig. 3B and C). Furthermore, connectivity density (a measure of bone quality) was significantly reduced in the fracture callus of r-mTBI mice (Fig. 3D). Together these data suggest that the newly formed bone in the fracture callus of r-mTBI mice has less mechanical strength. While FE analysis is an effective tool to study bone biomechanics, it has its own limitations [29]. Therefore, further studies using appropriate biomechanical testing on r-mTBI fracture bones are needed to validate the strength data predicted by the FE analysis.

It is noteworthy that previous studies reported that TBI, induced after weight drop trauma in our established model, leads to exuberant callus formation and fracture healing. In this regard, multiple clinical and preclinical studies have shown an enhanced callus formation and an increased callus volume in patients frequently reported to have concurrent TBI and bone fracture [16–18]. While increased fracture callus size has been observed in some animal models of TBI, we found that the amount of fully mineralized bone at the fracture callus is reduced nine weeks after fracture in the r-mTBI mice [18, 30]. One potential explanation for this difference is that in this study fracture healing was evaluated at 9 weeks post fracture when bone is completely remodeled, while in the published data, the fracture healing was evaluated after 3–4 weeks when the fracture callus is still undergoing remodeling. Second, the brain injury in our study was repeated for four days and bone fracture was performed one-week post r-mTBI, while in other studies the mTBI was performed only once and bone fracture was performed immediately after mTBI [18, 31].

In conclusion, our study shows that repetitive-mild trauma to the brain exerts negative effects on the healing of fractures over a long-term in mice.

## Limitations

(1) TBI occurs in both male and female veterans, but the prevalence of TBI has been reported to be higher in male veterans presumably caused by explosions or combat [32, 33]. It remains to be established if TBI symptoms are manipulated differentially in males and females due to sex hormone differences. In our study we chose male mice due to high prevalence of TBI in male Veterans. Further study with female mice needs to be tested to determine if the r-mTBI effect on fracture healing is gender-dependent. (2) As bone healing was evaluated only at the end of the study, additional time points are needed to evaluate the mechanisms for r-mTBI effects on impaired fracture healing. (3) We were not able to perform FE analysis in some of the bones as the boundary condition set to simulate the high friction compression test in the fractured callus did not work, thus reducing the sample size for this analysis.

## Data Availability

All data generated and analyzed in this study are included in this manuscript and are available from the corresponding author upon reasonable request.

## Abbreviations

r-mTBI: Repeated mild traumatic brain injury
BV/TV: Bone volume/total volume
CT: Computed tomography
PTA: Post-traumatic amnesia
CTE: Chronic traumatic encephalopathy
PTSD: Post-traumatic stress disorder
HPA: Hypothalamus pituitary axis
GH: Growth hormone
IGF-I: Insulin-like growth factor-1
Fig: Figure
G: Grams
Mg: Milligrams
mU: Milliunits
PDSII: Polydioxanone
kVp: Kilo volt peak
hrs: Hours
TV: Tissue volume
BV: Bone volume
N: Newton
MPa: Megapascal
EDTA: Ethylenediaminetetraacetic acid
ALP: Alkaline phosphatase
R: Correlation
FE: Finite element
